# Massive obesity and hyperphagia in posterior bilateral periventricular heterotopias: case report

**DOI:** 10.1186/s12881-016-0282-6

**Published:** 2016-03-09

**Authors:** Valeria Guglielmi, Roberto Floris, Monica D’Adamo, Francesco Garaci, Giuseppe Novelli, Paolo Sbraccia

**Affiliations:** Department of Systems Medicine, University of Rome “Tor Vergata”, Via Montpellier 1, 00133, Rome, Italy; Obesity Center, University Hospital Policlinico Tor Vergata, Rome, Italy; Diagnostic Imaging, University Hospital Policlinico Tor Vergata, University of Rome “Tor Vergata”, Rome, Italy; Department of Biomedicine and Prevention, University of Rome “Tor Vergata”, Rome, Italy; Medical Genetics Unit, University Hospital Policlinico Tor Vergata, Rome, Italy

**Keywords:** Bilateral posterior periventricular nodular heterotopia, Obesity, Hyperphagia

## Abstract

**Background:**

Bilateral posterior periventricular nodular heterotopia PNH is a complex malformation of cortical development with imaging features distinguishing it from classic bilateral PNH associated with *filamin* (*FLNA*) mutations. It distinctively consists of variably sized nodules of neurons along the trigones and temporal or occipital horns of the lateral ventricles and spectrum of developmental disorders of the mid-/hindbrain. This association suggests that pPNH is part of a more diffuse process of posterior or infrasylvian brain developmental abnormalities other than just a disorder of neuronal migration.

**Case presentation:**

This report describes the first case of an Italian young girl featuring pPNH and severe hyperphagic obesity. At the time of our first examination at age 3 years of age she was severely obese (body mass index, BMI 45.9 Kg/m^2^) and food-seeking behavior in the free-living situation was reported by the relatives. She showed normal linear growth and cognition, but mildly dysmorphic facial traits including deeply-set eyes, prominent zygomatic bones, downturned mouth corners and low-set ears. Over the years, the patient progressively developed further massive weight gain (at age 9 years, her BMI was 60.4 Kg/m^2^) and hyperphagia was confirmed by an *ad libitum* test meal. During follow-up, she presented limitations in walking capacity and in physical functioning due to the disabling obesity. On the basis of distinctive neuro-radiological findings pPNH was diagnosed, in absence of history of seizures.

**Conclusion:**

The present case may contribute to the expansion of the phenotypic expressions of this distinctive complex malformation.

## Background

Periventricular nodular heterotopias (PNHs) are common malformations of cortical development consisting of variably sized nodules of neurons along the lateral ventricles, associated with many clinical syndromes and different neuroimaging phenotypes.

Although traditionally PNHs have been considered the result of abnormal neuronal migration [1, 2] recent evidence has suggested that the primary cause may be a disruption of neuroependyma, which impairs postmitotic neurons from attaching to radial glial cells and, therefore, undermines initiation of the migration process [3, 4].

Patients with PNH represent a heterogeneous group from both genetic and phenotypic points of view. The classical bilateral X-linked form is usually caused by mutations of the *Filamin A* (*FLNA*) gene [5] and is characterized by bilateral symmetric heterotopic nodules along the frontal horns and bodies of the lateral ventricles. The clinical spectrum of *FLNA* mutations is wide and missense mutations or mosaicism may result in unilateral forms or nonlethal expression in males [6]. A rare autosomal recessive form is caused by mutations in the *ADP-ribosylation factor guanine nucleotide-exchange factor-2* (*ARFGEF2*) gene featuring microcephaly and delayed myelination in addition to bilateral PNH [7]. However, associations with several single-gene disorders, chromosomal anomalies [8] and disruptive causes [9, 10] have been reported, suggesting that heterogeneous genetic and nongenetic processes may cause PNH.

PNH can occur in different patterns and can be associated with different types of malformations. Indeed, they may either be found incidentally in asymptomatic patients, or more frequently discovered after imaging is performed for delayed development or epilepsy [11–13]. Fifteen subtypes have been so far identified on the basis of heterotopic nodules distribution and coexisting birth defects [14]. Bilateral posterior PNH (pPNH), the second most common variant after classic fronto-central PNH, refers to heterotopia exclusively located in the trigones and temporal or occipital horns of the lateral ventricles [15] and distinctively shows associations with a spectrum of infrasylvian developmental abnormalities.

## Case presentation

We describe the case of an Italian girl with severe early-onset obesity. She was second child of healthy, non-consanguineous parents. Gestation was uneventful but prenatal central nervous system ultrasound showed slight dilatation of the right lateral ventricle. Thus, the child was delivered at 38 weeks of pregnancy by Caesarian section and her birth weight and length were 3.17 kg (25–50th centile) and 48 cm (25–50th centile), respectively.

She presented neonatal hypotonia, delayed psychomotor development (she was able to sit without support at 18 months and started walking at 3,5 years) and rapid weight gain (her reported weight at 6 months was 15 Kg) with normal linear growth.

**Fig. 1:**
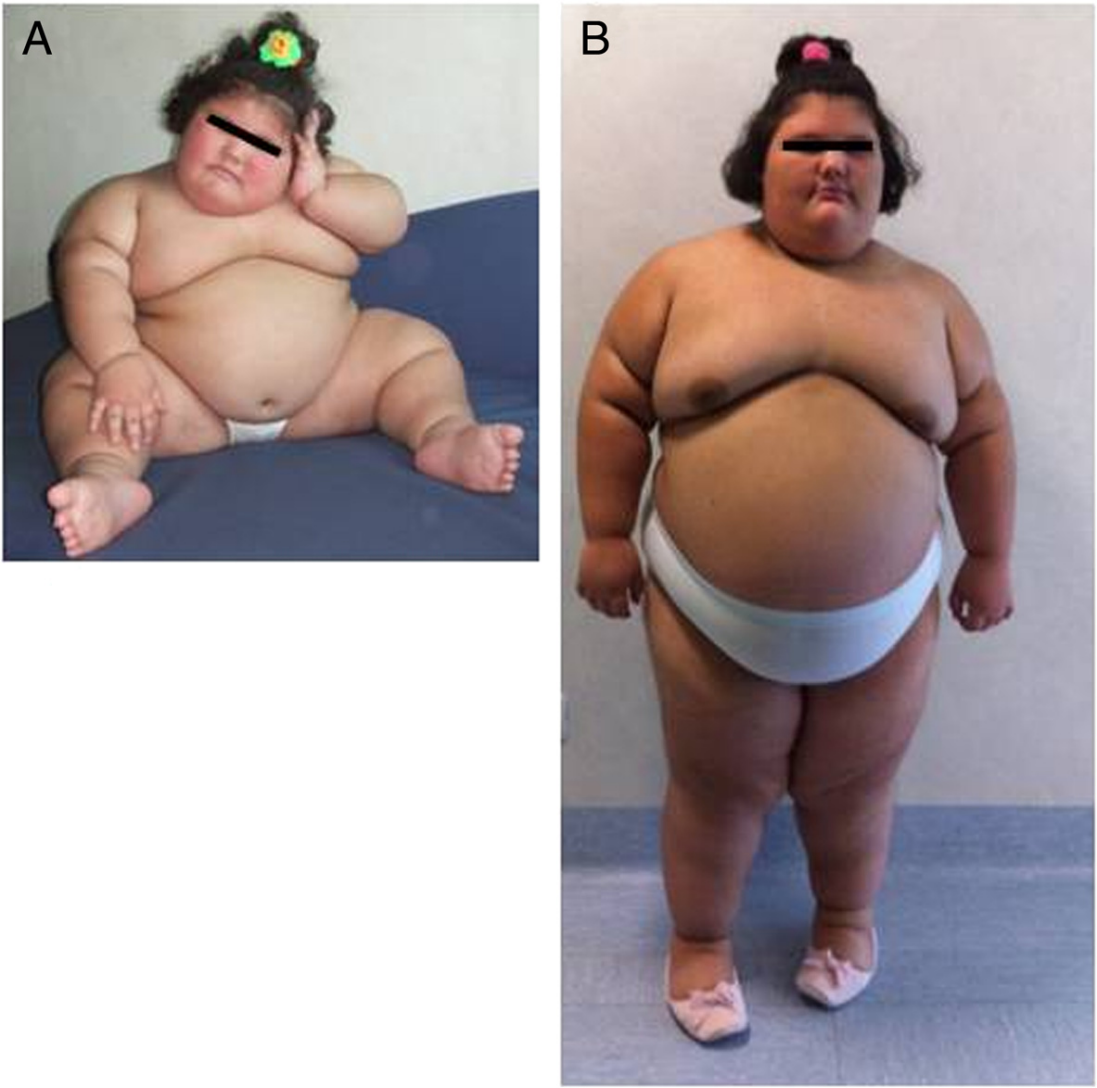
Patient’s clinical features. Patient at 3 (**a**) and 9 (**b**) years. Note the facial features, including prominent zygomatic bones and downturned mouth corners

At the time of our first examination she was 3 years old and severely obese: her body mass index (BMI) height and weight were 45.9 Kg/m^2^ (SDS-BMI: out of age range), 122 cm (SDS-height: 6.77) and 68 Kg (SDS-weight: 34.6), respectively (Fig. 1a). She was reported to be hyperphagic, showing food-seeking behavior in the free-living situation. There was no phenotype of morbid obesity in the parents or relatives.

Dysmorphic facial traits including deeply-set eyes, prominent zygomatic bones, downturned mouth corners and mildly low-set ears were also present (Fig. 1a-b). A comprehensive developmental assessment showed normal cognition, communication and motor skills. Ophthalmologic exam documented a mild left convergent strabismus but neither retinopathy, nor eye movement disturbances. Her blood pressure was in the normal range as well as electrocardiographic and echocardiographic evaluations. Radiologic assessment of the skeleton and hands showed no anatomical abnormalities.

The laboratory tests revealed hyperinsulinemia (36 μU/ml) and increased levels of liver enzymes (alanine aminotransferase -ALT- 41 UI/L; aspartate aminotransferase -AST- 73 UI/L; alkaline phosphatase -ALP- 334 UI/L; gamma glutamil transferase -γGT- 82 UI/L). Endocrinological assessment disclosed only a mild increase in adreno-corticotropic hormon (ACTH: 92 pg/ml) with normal serum cortisol levels (13 μg/dl) in line with the degree of obesity. Clinical signs of insulin resistance (acanthosis nigricans of the neck and armpits) and hepatomegaly (also confirmed by abdominal ultrasound scan) were also detected.

She had premature pubertal development and menarche at seven years of age, with post-puberal gonadotropins and sexual hormones levels.

She progressively developed further massive weight gain and, at the time she was nine years old, her BMI, height and weight were 60.4 Kg/m^2^ (SDS-BMI: 13.6), 145 cm (SDS-height: 2) and 127 Kg (SDS-weight: 23.2) respectively (Fig. 1b). Hyperphagia was confirmed by an 8.2 MJ energy intake in an 18 MJ *ad libitum* test meal. The degree of hyperphagia (120.6 KJ/Kg lean mass) was comparable to that reported in 50 six-year old patients with heterozygous mutations in the melanocortin 4 receptor gene [16] and in one patient with congenital deficiency of prohormone convertase 1/3 [17]. Basal metabolic rate measured by indirect calorimetry after an overnight fast was comparable (8.2 MJ/d) to that predicted by age and gender-specific equations (8 MJ/d) [18].

During an abdominal ultrasound reevaluation, a 25 cm simple ovarian cyst was also detected.

She presented severe impairment of walking capacity and worsening limitations in physical functioning due to her disabling massive obesity. In her primary education, mild intellectual disability appeared as she was perceived by her teacher as having special educational needs, so that she started receiving an inclusive classroom program.

**Fig. 2:**
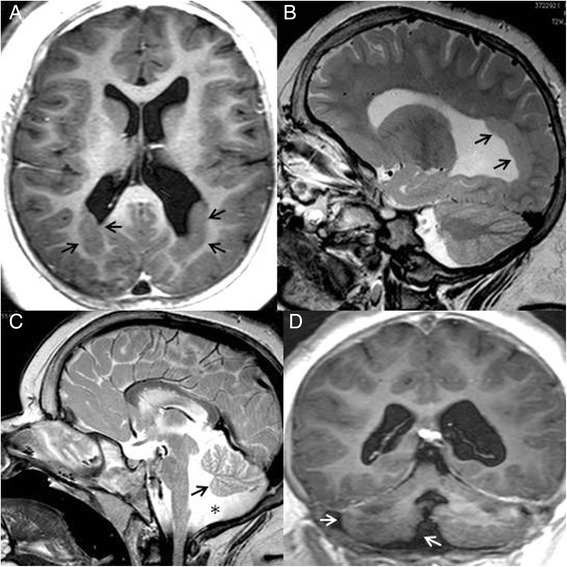
Brain Magnetic Resonance findings. Axial T1 weigthed image (**a**) and sagittal T2 weighted image (**b**) show PNH lining the wall of trigones and occipital horns of lateral ventricles (*arrows* ↗) and asymmetric colpocephaly. Sagittal T2 weighted image demonstrates small cerebellar vermis (*arrows* ↗) and megacisterna magna (*asterisk* *) (**c**). Coronal T1 image shows hypoplasia of right cerebellar hemisphere (*arrows* ↗) (**d**)

Brain Magnetic Resonance Imaging (MRI) showed complex developmental abnormalities consisting of bilateral symmetric nodules of grey matter lining the walls of the trigones and occipital horns of lateral ventricles colpocephaly, transmantle bands extending from the posterior nodules to the overlying dysplastic cortex, hippocampal malrotation, cerebellar hemispheres asymmetry, vermis hypoplasia with large cisterna magna and missing of the normal T1 hyperintensity of neurohypophysis (“bright spot”) (Fig. 2). On the basis of these neuro-radiological findings pPNH was diagnosed.

An EEG, performed for the concern of seizures, showed normal findings with mild and diffuse non-specific anomalies.

Genetic testing was performed. The high resolution karyotype analysis and the array comparative genomic hybridisation (CGH) were negative. Neither deletions and duplications of the whole locus, nor point mutations in the coding exons of *FLNA* gene were detected. Additionally, DNA methylation analysis within the chromosome 15q11.2-q13 excluded the Prader-Willi syndrome.

## Conclusion

The first case featuring pPNH and severe hyperphagia/obesity is herein reported.

Patients with pPNH are characterized by nodules typically located along the atria and temporal horns of the lateral ventricles and developmental disorders of the mid-/hindbrain. This association suggests that pPNH is part of a more diffuse process of posterior or infrasylvian brain maldevelopment other than just a disorder of neuronal migration [15].

Cerebellar signs ranging from severe cerebellar syndrome to mild dysmetria, nystagmus and dysarthria were reported in patients affected by pPNH. Focal epilepsy and normal to mildly impaired cognitive level were also documented [14, 19]. The severe ambulation impairment observed in our patient was not ascribed to cerebellar abnormalities but to the disabling obesity. Besides, our patient did not present either mental retardation or history of seizures.

In keeping with the marked reduction in white matter volume observed in pPNH, some authors hypothesized that this pattern presents greater frequency of connectivity abnormalities [20]. Given also that in Prader-Willi syndrome altered functional connectivity alterations among the brain regions implicated in eating and rewarding have been reported [21], it would be intriguing to speculate that in pPNH some theoretical neural pathways that control feeding behavior might be disrupted. However, we failed to observe other signs of hypothalamic dysfunction such as temperature instability, high pain threshold, sleep disordered breathing and endocrine abnormalities. Neuro-radiologic alterations of hypothalamus anatomy were excluded as well.

Previous observations have demonstrated that PNH is clinically and genetically heterogeneous and causative genes appear to be scattered through the genome. Whereas the classical bilateral X-linked form is usually caused by mutations of the *FLNA* gene involved in regulation of cell stability, filopodial protrusion and motility across various biological systems, in contrast, the genetics of pPNH is so far unknown. In fact, the posterior pattern of PNH does not harbour mutations in *FLNA.* Accordingly, in our patient no *FLNA* mutations were detected. Thus, pPNH may be considered as a specific malformation complex, which entails a more extensive disturbance in cortical development and putatively reflects aberrations in different mechanisms or pathways to classic PNH. The association of pPNH with mid-/hindbrain malformations may suggest that genes expressed more strongly in the posterior telencephalon might be likely causative candidates [20] in this pattern of PNH.

The hyperphagic behavior and massive obesity observed in our patient represent a novel clinical feature in pPNH. Although the nosologic classification of pPNH is inherently limited by the lack of genotype-phenotype correlations, and a concomitant monogenic cause (e.g. melanocortin 3 or 4 receptor, leptin and leptin receptor mutations) of the hyperphagic behavior cannot be ruled out, our report reinforces the clinical, and putatively genetic, heterogeneity of pPNH. After all, some of these mutations appear not very likely on the basis of the clinical findings. Namely, disorders in leptin production or action result, beyond severe early-onset obesity, in altered immune function due to T-cell defects and pubertal delay [22], the latter being in conflict with the premature pubertal development and menarche of our patient. Similarly, children with mutations in the neuroendocrine-specific enzyme prohormone convertase 1/3 present early-onset obesity, reactive hypoglycemia, enteropathy, impaired proopiomelanocortin processing (with consequent decrease in ACTH plasma concentrations) and neuroendocrine dysfunction [23], features that were not present in our patient. Rather, we found a mild increase in ACTH levels (considered in line with the degree of obesity).

In conclusion, we believe that the present case may contribute to the expansion of the phenotypic expressions of this distinctive complex malformation. However, this case could also represent a new disease entity.

## Consent

Written informed consent was obtained from the minor child’s mother for publication of this Case report and any accompanying images. A copy of the written consent is available for review by the Editor of this journal. Clinical tests performed and blood sample collection conform to the routine standard care.
